# A Review of Computational Methods to Predict the Risk of Rupture of Abdominal Aortic Aneurysms

**DOI:** 10.1155/2015/861627

**Published:** 2015-10-05

**Authors:** Tejas Canchi, S. D. Kumar, E. Y. K. Ng, Sriram Narayanan

**Affiliations:** ^1^School of Mechanical and Aerospace Engineering, Nanyang Technological University, 50 Nanyang Avenue, Singapore 639798; ^2^Lee Kong Chian School of Medicine, Nanyang Technological University, 50 Nanyang Drive, Singapore 637553; ^3^Department of General Surgery, Tan Tock Seng Hospital, 11 Jalan Tan Tock Seng, Singapore 308433

## Abstract

Computational methods have played an important role in health care in recent years, as determining parameters that affect a certain medical condition is not possible in experimental conditions in many cases. Computational fluid dynamics (CFD) methods have been used to accurately determine the nature of blood flow in the cardiovascular and nervous systems and air flow in the respiratory system, thereby giving the surgeon a diagnostic tool to plan treatment accordingly. Machine learning or data mining (MLD) methods are currently used to develop models that learn from retrospective data to make a prediction regarding factors affecting the progression of a disease. These models have also been successful in incorporating factors such as patient history and occupation. MLD models can be used as a predictive tool to determine rupture potential in patients with abdominal aortic aneurysms (AAA) along with CFD-based prediction of parameters like wall shear stress and pressure distributions. A combination of these computer methods can be pivotal in bridging the gap between translational and outcomes research in medicine. This paper reviews the use of computational methods in the diagnosis and treatment of AAA.

## 1. Introduction

Rapid improvements in computational power coupled with better understanding of hemodynamics have spawned the interdisciplinary science of computational medicine. A multidisciplinary effort with clinicians, radiologists, and biologists on the one hand and engineers and computer scientists on the other hand has greatly increased the ability to diagnose medical conditions and has improved delivery of healthcare. Computational methods have played a very important role in this, as experimentally determining parameters that affect a certain medical condition is not possible in many cases. CFD methods can be used to accurately determine the nature of blood flow in the cardiovascular system [1] and the nervous system [2] and air flow in the respiratory system [3]. Machine learning/data mining methods have also been used to develop models that learn from retrospective data to make a prediction on factors affecting progression of disease [4–6]. These models have also been successful in incorporating factors such as patient history and occupation. Some of these variables (patient history, occupation, and family history) are difficult to quantify directly and so, using methods such as machine learning, they can be incorporated into predictive models.

**Figure 1:**
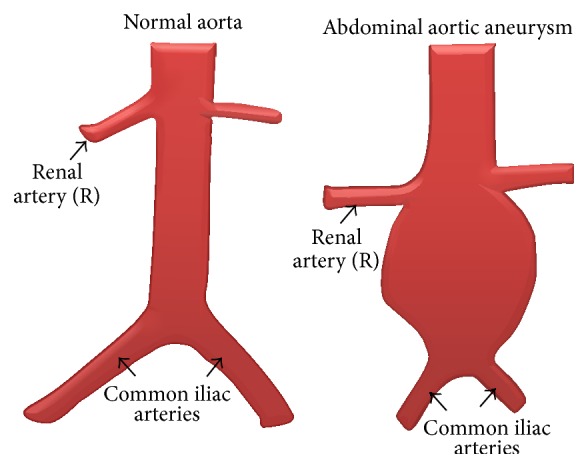
Abdominal aortic aneurysm.

AAA is a condition affecting the aorta usually in its infra-renal segment and involves the abnormal dilatation of this artery (Figure 1). The infrarenal aorta is a site predisposed to aneurysmal widening. The cyclic stress caused by the pulse wave in conjunction with factors which decrease the strength of the wall may lead to dilatation and ultimately to rupture [7]. It is the 13th most common cause of death in the United States [8]. The condition occurs mainly in patients over 65 years of age and affects approximately 2% of the elderly population [9]. There are several risk factors that have been known to affect the genesis, growth, and rupture of AAA: advanced age, greater height, coronary artery disease, atherosclerosis, high cholesterol levels, hypertension [10], and smoking [11, 12]. AAAs are known to have an incidence that is approximately four to six times higher in men than in women. However, the incidence in women also rises with age, although it starts later in life than in men [13].

Surgical repair of AAA can be performed in two ways, by traditional open surgery or endovascular aortic aneurysm repair (EVAR). Initially, open surgery was the norm but since the introduction of EVAR [14], there has been a widespread acceptance of the procedure. This is a less invasive procedure with a decrease in 30-day mortality in patients when compared to open surgical repair. In addition, the current generation of devices approved has smaller delivery system profiles, tracks better, and can be used to treat difficult AAA morphology more easily [15]. EVAR has rapidly become the preferred method of AAA repair amongst vascular surgeons. Vascular surgeons have used lumen diameter as a metric to guide surgical intervention for some time now. A cutoff diameter of about 5–5.5 cm is used as a base to recommend surgery for patients with AAA [16]. Some studies like The UK Small Aneurysm Trial Participants, 1998 [17], have reinforced this value as a reasonable measure of the risk to benefit ratio between the risks of aneurysm rupture and those of surgical intervention.

As a criterion, the use of the maximum diameter metric is a crude way to estimate the critical state of an AAA [18]. Vorp et al. [18] argued that, from a biomechanical perspective, the use of wall stress in the lumen can more accurately predict the rupture of AAA. They defined the critical state of an AAA as that at which the mechanical stress within the aneurysmal wall exceeds the tensile strength of the tissue. Other parameters such as wall tensile strength, length of aneurysm, and patient-specific pulsatile velocity and pressure boundary conditions also play an important role in the progress to rupture of the AAA. Biomechanics of rupture in AAA is affected due to variations in a combination of these parameters. Hence, to account for the contributions of several parameters in what is essentially a patient-specific problem, computational methods could play a crucial role. This paper provides a summary of the different computational methods that can be used, a review of the literature that has incorporated computational methods to account for several parameters that lead to rupture in AAA, and suggestions on the direction that future research using these methods should take in order to improve our understanding of the rupture of AAA. Computational methods in imaging are left out of this review as the focus is on the biomechanical analysis of AAA.

## 2. Computational Methods

### 2.1. CFD Methods

**Figure 2:**
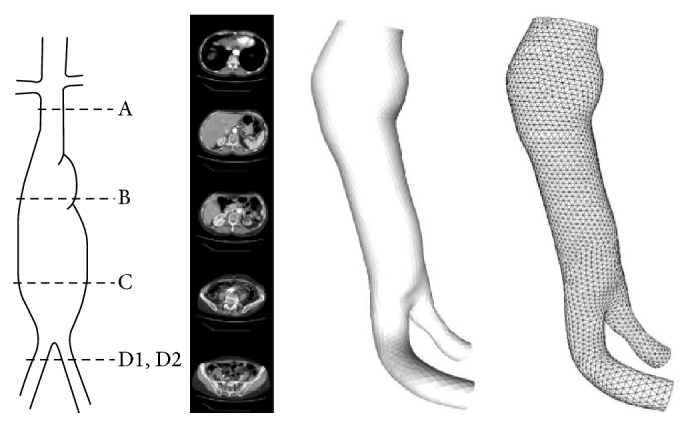
AAA geometry from CT images [19].

Biomechanical analysis of an AAA system involves a series of steps to assess the factors that may influence the risk of rupture. Firstly, preoperative computed tomography (CT)/magnetic resonance imaging (MRI) images of the diseased aorta are obtained. These are then put through a process of image segmentation to convert what are 2D slices into a 3D geometric model, suitable for use in a CFD code (Figure 2). The pulsatile velocity and pressure boundary conditions for use in the CFD model are obtained during surgery. These are measured at the inlet and outlet of the aneurysm and at any other suitable point of interest for the researcher. CFD codes are then used to obtain the wall shear stress, pressure distributions, and the flow physics of blood in the aneurysm.

Computational methods in biomechanics incorporate the parameters mentioned in the previous section. Models are based on constitutive laws of continuum mechanics, such as the Law of Laplace that had been originally used in the analysis of bursting of cylindrical shells, the laws of rheology to describe the properties of blood, fluid mechanics in the analysis of vortex formation in the lumen, and material properties of stent grafts in a postsurgical aorta. Most CFD solvers are based on the Navier-Stokes (N-S) equations (1) which form the basis for describing the flow of fluids. They describe the momentum field of the flow under investigation and need to be used in conjunction with the continuity equation that describes mass transport

(1) ∂ui∂xi=0,ρ∂ui∂t+uj∂ui∂xj=−∂P∂xjδij+μ∂2ui∂xj∂xj+fi,

where *u* is the velocity, *P* is the pressure and *ρ* is the density of the fluid and *f* is the body force acting on the fluid. The N-S equations are discretized using two methods: the finite volume method (FVM) and the finite element method (FEM). FVM is generally seen to approximate the solution accurately to a large extent. But FVM may not be the best way to understand the deformation of a tissue due to blood flow. FEM has been used to solve fluid mechanics based problems [20, 21] and specifically for AAA simulations as well [22, 23]. It has the capability to incorporate the displacement of the biological tissue in a medical simulation problem more accurately than a FVM-based solver where the fluid becomes the most important component in the solution unlike in FEM where the solid displacement is more important.

The most accurate solutions to a biomechanics problem would incorporate the material properties of the blood vessel/tissue in question. This would allow a fluid-structure interaction (FSI) model to be embedded in the resulting physics. As the blood vessel/tissue is normally not rigid, it is imperative that this aspect is accounted for in the computed solution. FSI methods [24, 25] have been demonstrated to be effective in the description of flow physics in aneurysms. The coupling of the aneurysmal wall motion and blood flow is commonly made by using the Arbitrary Lagrangian Eulerian (ALE) method. The incompressible continuity and N-S equations in ALE form can be expressed as

(2) ∇·u=0ρf∂u∂t+u−ug·∇u=−∇p+μ∇2u,

where *ρ*_*f*_, **p**, **u**, and **u**_*g*_ are the fluid density, the pressure, the fluid velocity, and the moving coordinate velocity, respectively. In ALE formulation, the term (**u** − **u**_*g*_), which is the relative velocity of the fluid with respect to the moving coordinate velocity, is added to the conventional Navier-Stokes equation to account for the movement of the grid [26]. From a computational point of view, we have to incorporate the moving interface between the fluid and the rigid body. The ALE method has been successfully applied to such moving boundary problems, which arise in free surface problems and fluid-structure interaction problems.

The ALE method has been employed because

- it is convenient to describe the fluid motion on the moving interface by the Lagrangian description in order to treat the compatibility conditions and the equilibrium conditions between the fluid and the rigid body;
- it is apparently impossible to employ the Lagrangian description for the entire fluid domain because of severe mesh distortion due to vortex shedding or flows through outer boundaries.

Therefore it is natural to employ the mixed viewpoint of the Lagrangian and Eulerian descriptions [27]. Whilst the above method has been used for a rigid tissue, the ALE has been extended to the FSI problem as well [28]. Hirt et al. provide further detail of the ALE method [29] wherein they describe the applications of ALE to problems involving different flow speeds using the finite element method.

### 2.2. Machine Learning Methods

Significant experience over many years in the management of AAA amongst hospitals and clinicians has led to the availability of a large volume of data on patients who have undergone treatment for the condition. Statistical techniques such as univariate and multivariate logistic regression analyses have been successfully applied to risk prediction in clinical medicine. A commonly used instrument is the use of a prognostic score derived from logistic regression to classify a patient into a potential risk category [30]. This suggests that, using these techniques, an estimate of the associations between the various risk factors that cause rupture in an AAA can be encoded.

Many techniques have been used for data mining in medical and biomedical studies. A popular data mining technique is decision tree induction. The dataset is recursively partitioned into discrete subcategories. These subcategories are based on the value of an attribute in the dataset. The criteria for selecting these attributes in the dataset are based on its predictability within a certain subcategory. As a result of this, the final outcome is a set of series of categories based on values of the attributes. Each of these series generates a classification value. There are many algorithms developed for decision tree induction. Some clinical examples that apply this approach are diagnosis of central nervous system disorders [31], posttraumatic acute lung injury prediction [32], and acute cardiac ischemia [33].

Some of the parameters that may affect the ability to predict rupture risk in an untreated aneurysm are listed here. These parameters can be used in a model that learns from retrospective data and can be used in a prospective tool for patients.

1. Diameter of lumen.
2. Length of aneurysm.
3. Aneurysm neck angle and length.
4. Wall thickness.
5. Presence and volume of intraluminal thrombus (ILT).
6. Wall shear stress (WSS).
7. Calcification of the aortic wall and calcium volume and percentage of aneurysm sac volume.
8. Gender.
9. Age of patient.
10. Patient history (smoking, diabetes, specific occupations, and family history of aneurysms).
11. CT scan slice thickness.
12. Aortic and iliac vessel tortuosity.
13. Imaging system used for diagnosis.
14. AAA ILT index.
15. Patient ethnicity.
16. Body mass index (BMI).
17. AAA surface area.
18. Aneurysm neck length/height and supra- and infrarenal neck angles.

Whilst these may be some of the input variables for a machine learning model, the output is the risk of rupture that can be obtained as a result of training the model on such retrospectively gathered data. The said model will learn about the parameters that play a part in the rupture or nonrupture of a patient-specific AAA. A great amount of classified data, that is, a suitable spread of ruptured and nonruptured retrospective cases, will allow the model to gauge the parameters that are the most important factors among many that lead to rupture in an AAA case. This model can then be applied prospectively where the model, having learnt from a large amount of data in the past, can to a degree of accuracy make a prediction of rupture risk in the patient.

When comparing different classifiers [34, 35] the key issues to address in such a model are

1. predictive accuracy;
2. interpretability of the classification models by the domain expert;
3. handling of missing data and noise;
4. ability to work with different types of attributes (categorical, ordinal, and continuous);
5. reduction of attributes needed to derive the conclusion;
6. computational cost for both induction and use of the classification models;
7. ability to explain the clinical decisions made when models are used in decision making;
8. ability to perform well with hitherto unseen (prospective) cases.

## 3. Computational Methods in AAA Analysis

The risk of rupture of an AAA has been studied in several different ways. Some researchers have performed experiments on phantoms made of silicone rubber [36] and distensible tubes [37–39], on cadavers, and during surgery to ascertain the properties of the AAA configuration [18]. Some of them have come up with analytical approaches of a simplified AAA problem [40–42]. But CFD approaches have dominated the landscape in the analysis of risk of rupture of AAA. Experiments are difficult to set up due to ethical considerations of sample retrieval from patients. Material properties need to be accurately obtained for the simulation to be of any use clinically. This has been a major challenge in AAA rupture studies.

### 3.1. Boundary Conditions

**Figure 3:**
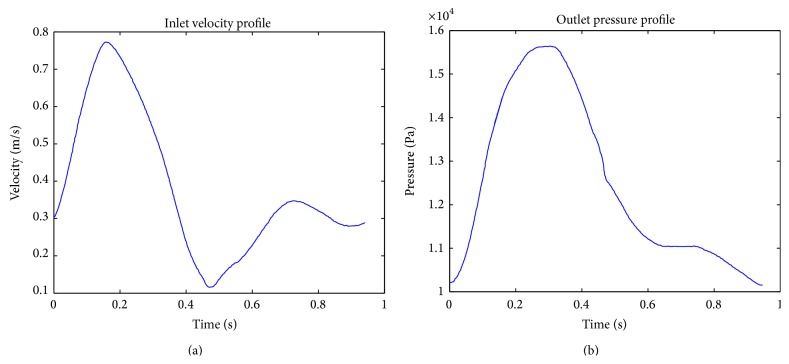
Inlet velocity and pressure outlet waveforms [43].

Unsteady boundary conditions of velocity and pressure are generally obtained from phase contrast MRI methods. There is a paucity of such studies and, in most instances, a few waveforms that are available in literature are reused by others, thereby taking away the patient-specific nature of the problem being solved.  Figure 3 shows such a sample waveform that has been used by Soudah et al. [43], which in itself has been adapted from Ouriel et al. [9].

Flow in the aorta is pulsatile in nature. Hence, time varying waveforms of velocity and pressure are needed in order to accurately model the flow physics. The inlet flow velocity profile has two peaks as seen in Figure 3, each visualizing the systolic and diastolic phases in a pulse. The outlet pressure shows only one peak in the pulse.

Several different kinds of outlet boundary conditions have been used in literature such as constant pressure [44, 45] and flow specification at particular instances [46]. The boundary conditions for the velocity *V* are imposed as follows: (i) no slip at the walls, (ii) uniform (slug) profile at the inlet, and (iii) zero-traction outflow at the exit. For pulsatile flow, the inflow mean velocity is time dependent and the volume flow rate is oscillatory as suggested by Finol and Amon, 2003 [47]. Although newly developed imaging methods, such as 4D MRI, have proved to be successful in measuring the flow, they still have limited spatial resolution (of the order of 2 mm), which restricts their applicability in calculating WSS parameters, and do not provide pressure information. Therefore, in the usage of Windkessel parameters based on patient-specific clinical data, the simulation takes into account the essential characteristics of the rest of the vasculature, which can be expected to improve the correlation between the simulation results and the true in vivo characteristics [48].

The Womersley number is a nondimensional number that describes the ratio of pulsatile flow to viscous effects and is used extensively in computations related to biofluids. Womersley profiles generally regard only the centerline velocity of the inlet. They incorporate the Bessel functions to derive the velocity profile in the inlet. But, it is not accurate as the geometry of the artery is patient-specific and affects the velocity values. The Womersley number is as shown below [49]:

(3) $$\begin{array}{c} \alpha =r{\left(\;\frac{\omega }{v}\;\right)}^{1/2}, \end{array}$$

where *α* is the Womersley number, *r* is the radius of the artery, *ω* is the angular frequency of flow oscillation, and *υ* is the dynamic viscosity of the fluid.

Flow boundary conditions are critical to computing the flow solution in AAA. Hardman et al. [49] surmise that the Womersley number-based studies are inaccurate in computing solutions of flow fields and hence an axial velocity component is needed. They suggest that it is sufficient for the axial component to be used in the simulation and radial components are needed only when it is needed to understand the secondary flow components in the flow, such as particle tracking. Multiple outlet systems are, however, more complex and need better description of boundary conditions. Though this seldom occurs in the case of AAA, scalability, simplicity, and accuracy are paramount as there could be multiple outlets in an arterial network [50].

### 3.2. Material Properties

There are other boundary conditions to be considered in the simulation of AAA fluid mechanics. These are the material properties of the arterial wall. This requires the arterial wall material to be properly modeled, an issue which is complex given that the wall stiffness increases when lumen diameter increases and calcification and medial sclerosis occur with aging and in disease. Furthermore, the aneurysmal wall has been found to be mechanically anisotropic, a factor to be taken into account if a rational estimate of the wall stresses is to be made [51].

Generally, an isotropic properties assumption is made of the arterial wall when carrying out an analysis of the AAA. Whilst this may be a reasonable assumption in most cases, a more accurate constitutive model is needed to describe the properties of the wall. Constitutive models are obtained from experimental tests of actual tissue specimens. A uniaxial test specimen is generally made from two rectangular strips of tissue that has been cut out from the arterial layer. The flat arterial tissue layer is assumed to be a fiber-reinforced material with relatively stiff collagenous fibers embedded in a homogeneous isotropic (soft) ground matrix [56].

Most elastic tissues studies in literature have used the strain energy function approach. But arterial tissue exhibits highly nonlinear, nonisotropic, and possibly hyperelastic properties, and a computational model needs to incorporate all these properties as the elastic modulus is inadequate. Also, the assumption of strain energy functions holds good only for single continuous medium tissues. This is not the case with arterial tissue [57]. Since the mechanical properties of soft biological tissues depend greatly on their microstructure, proposing a reliable mechanical model for these tissues, including the arterial wall, depends on the level of microstructure integration attained in the constitutive model [58]. Taghizadeh et al. [58] proposed a new biaxial constitutive model based on microstructural properties as opposed to the simple uniaxial tests carried out by Sokolis et al. [59] and Karimi et al. [60]. The uniaxial tests only look at a single layer of the tissue that is in contact with the blood flow. The biaxial tests however consider a second layer as well, which is important in assessing the response of the arterial wall in a fluid-structure interaction scenario. In general, whilst using the simplified boundary conditions is computationally inexpensive and is accurate in most cases, incorporation of the nonlinear properties when custom codes are written makes the solution more accurate.

Wall thickness is another aspect that is central to the response of the blood vessel to flow. Since aneurysmal rupture occurs at a specific site of the aortic lumen, the properties of the wall affect the computed solution. This is because the wall thickness is seen to reduce as the disease progresses. In general, the aortic wall thickness in computational methods is assumed to be in the range of 1.5–2 mm. While this may be largely accurate for most simulations, it has been acknowledged as a major limitation in the completeness of the prediction solution [61]. Some previous measurements of thickness are listed in Table 1 [52].

The thickness from Thubrikar et al. [55] has been extensively used in literature as the value that accounts for the posterior and anterior sections of the AAA. Most ruptures are seen to occur in the proximal posterior part of the AAA. Hence, this value is assumed to accurately describe the wall thickness.

Raut et al. [52] suggest that the wall thickness as a constant value is not accurate and described a novel method that incorporated the regionally varying wall thickness, especially in the area of rupture. A comparison of uniform thickness, patient-specific uniform thickness, and the varying thickness was carried out in 28 samples. This showed a statistically significant difference on FE analysis with principal stresses and strains and strain energy density being the output parameters. As stated previously, rupture does not occur at the region of the aortic wall with maximum lumen diameter. This method can thus be used to obtain the carrying wall thickness that reflects the true thickness at the site of rupture.

### 3.3. Flow Physics

The physics of blood flow through the AAA is perhaps the most important aspect of the analysis of rupture risk. Data about material properties from experimental studies and boundary conditions from advanced MRI and CT technology have led to CFD methods being used to understand the flow fields in AAA. Accurate hemodynamic simulations can lead to better treatment planning and improved stent design for the diseased aorta. The interaction between blood flow and the arterial wall is a challenging problem. The studies reported below have used the rigid wall assumption to overcome the computational expense of the fluid-structure interaction solution.

Blood flow in the normal aorta can be ascribed to be laminar, similar to that in a pipe. Flow development is also well understood. In contrast to the normal aorta however, the flow in an aneurysmal segment is highly disturbed and maybe nonlaminar. Specifically in the aortic segment immediately beyond the aneurysm neck, flow separation involving regions of high streaming velocities and high shear stresses is observed [62]. At the expanded aneurysmal segment, average flow velocity and wall shear stress are much lower compared to those in the normal aorta [62]. Early studies used steady flow computations as the norm as pulsatile boundary conditions were hard to obtain. Taylor and Yamaguchi in 1994 [63] were amongst the earliest to compare the steady and unsteady flows in aneurysms. A set of symmetric vortices that were different in behavior downstream were observed in both the steady flow and the unsteady flow conditions. Symmetric vortices induce high pressure at certain locations in the lumen and were seen later on to be possible sites of rupture of the aneurysm.

Blood flow induces wall shear stress (WSS) in the aneurysm. This is the main parameter that determines the risk of rupture in an AAA lumen. WSS is related to the properties of the blood flowing through the lumen. Apart from blood pressure, WSS in AAA is also influenced by the aneurysm diameter, shape, wall thickness, wall mechanical properties, and the presence of intraluminal thrombus (ILT) [64].

ILT is an important component of AAA that is difficult to incorporate into any computational model. Most aneurysms have ILT within their lumen. Stenbaek et al. [66] argued that the development of ILT may be a better predictor than the maximum diameter of the AAA as a rupture risk parameter. But Di Martino and Vorp [67] postulated that ILT might protect the AAA wall from the pressure applied by blood flow. A finite element study by Li et al. [68] on the effect of ILT on wall shear stress showed that the non-ILT models had higher stress development than the ILT models. This is in keeping with Laplace's Law, which is often applied in fluid mechanics studies on AAA. It must however be emphasized here that Laplace's Law was originally used to describe bursting stresses in cylindrical shells and to apply it in this situation may not be wholly accurate. Thus, the role of ILT in the biomechanics of AAA is yet to be ascertained clearly. This might be because of the different types of ILTs that develop inside the AAA. O'Leary et al., 2014 [69], performed mechanical tests on 356 samples and classified them into 3 morphologies, type 1 which was a multilayered ILT whose strength and stiffness decreased gradually, type 2 whose strength decreased abruptly, and a single layered ILT with lower strength and stiffness compared to the other two types. This may partially explain why there is a difference of opinion amongst researchers about the effect of ILT in AAA. There are differences that crop up in ILT even due to gender. This aspect has been brought out by Tong et al. in 2013 [70] when they carried out biomechanical behavioral studies on 90 AAA samples (78 men and 12 women). They observed that the female ILT luminal layer showed a lower stiffness in the longitudinal direction than the males and, consequently, the thrombi may have different wall weakening effects in males and females.

While most methods have assumed laminar flow regimes in the aneurysm, there is a possibility that turbulent flow can also develop due to the presence of ILTs or wall calcification. Turbulent flow is common in the heart and the upper airway unlike the smaller diameter aorta where a discernible turbulent regime of flow is not seen. However, turbulence may well be a factor in very large aneurysms where there is the possibility for a turbulent mixing phenomenon to occur. In a study using a rigid wall Newtonian fluid approach considering turbulent conditions carried out on 3 symmetric aneurysm geometries with different diameters, the smallest aneurysm did not show transition while the biggest diameter aneurysm shows flow separation and turbulent vortices [71]. It was seen that the vortex that impinges on the distal wall of the AAA generates secondary vortices, which then break up further. In the small aneurysm where flow remained laminar throughout the cycle, the high stress region was only seen in a relatively thin layer attached to the aortic wall. The large aneurysm, however, was seen to have islands of high stress in the middle of aneurysm due to the impact of turbulence transition [71]. This study on the impact of turbulence transition on WSS concluded that, with the onset of flow turbulence as seen in the large aneurysm models, the surface stress appeared to point to all directions and no dominant direction could be identified. The temporal and spatial gradient of WSS is significantly larger than the laminar flow (small aneurysm) situation. It is however important to consider the effect of turbulence as well which is seen to occur in larger aneurysms, especially above 5 cm. Given the inherent nature of flow, the transition to turbulence can occur even at resting conditions in large aneurysms.

### 3.4. Fluid-Structure Interaction (FSI)

In computed solutions where the aneurysmal wall is assumed to be rigid, pressure values do not vary in time inside the lumen as the wall and blood flow are not coupled in the solution. But, in reality, the wall is not rigid and interacts with the flow during every pulse. The nature of this fluid-structure interaction is bidirectional; that is, the wall responds to blood flow pressure, shown by its displacement, and this in turn affects the flow through the aneurysm. FSI methods most accurately describe the flow physics occurring inside the aneurysm and in theory can precisely predict the site of rupture with the wall shear stress values reflecting the most realistic conditions.

**Figure 4:**
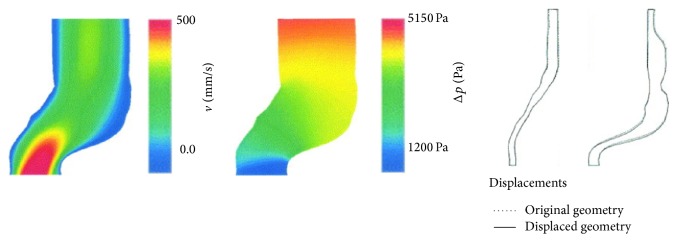
Velocity and pressure distribution along with wall displacement in a FSI calculation. (Adapted from Di Martino et al., 2001 [65].)

Di Martino et al. [65] first carried out a FSI calculation on a realistic geometry obtained from segmentation of CT images (Figure 4). They were able to establish local stresses on the wall due to the structural and blood flow conditions. An ALE based FSI numerical method was used in commercial software Fidap to obtain the solution. The motion of the wall was not homogenous as would be seen in a rigid wall calculation. The asymmetry of the aneurysm also seemed to play a part in the development of wall stresses. They found that a larger area of maximum velocity corresponds to the systolic peak and that a flow deceleration is always discernible at the site where the aneurysm is larger, suggesting a higher pressure at these sites. This along with a weakening of the wall itself may be the genesis of continued aneurysm enlargement.

These studies have shown that a FSI method would be most accurate in predicting wall motion in the aneurysm as against only a FEM or a fluid dynamics model [75, 76]. This was further demonstrated by Scotti et al., who compared the FEM with a FSI method on 10 aneurysm models. It was observed that applying a realistic fluid pressure distribution to the arterial wall resulted in wall stresses that were 20% higher than if only peak systolic pressures were considered. This was in direct correlation with previous results that also showed a 21% increase in wall shear stresses when FSI methods were used [73, 77]. A limitation of this study was that this was an idealized model with asymmetry built into the geometry.

Using FSI methods, the various geometrical changes and material characteristics in patient-specific aneurysms can be accounted for. Scotti et al. compared aneurysm geometries having uniform wall thickness and variable wall thickness and also investigated the effect of 5 levels of asymmetry in the model. They observed that varying wall thickness increases the von Mises stress by up to 4 times as compared to when it is uniform. For asymmetrical variations too, the variation of thickness caused stresses to increase. This reinforces the fact that accurate thickness and FSI considerations will make the computed solution more accurate.

Li and Kleinstreuer [74] reinforced this by comparing different asymmetrical aneurysm geometries in a FSI solver. They concluded that assumption of symmetry leads to underestimation of wall shear stress. An iliac bifurcation angle also seems to have an effect. For iliac bifurcation angles less than 90 degrees, the wall shear stress is in the range of 0.57 to 0.63 mPa. But as it goes beyond 90 degrees, the stress increases by about 8%. At aneurysm neck angles of 12 degrees or so, the region with the higher wall stress is at the asymmetric bulge with the maximal stress being at the distal point of the bulge. As the neck angle increases, the proximal stresses move away from the bulge but the maximum point of stress remains the same. This, they argue, is due to the fact that neck angles influence strong surface curvatures in the AAA neck leading to changes in the location of wall stress regions.

A summary of the different studies using FSI methods is listed in Table 2.

### 3.5. Machine Learning Methods

As described in the previous section, fluid-structure interaction methods greatly increase accuracy of simulations of AAA, leading potentially to more accurate surgical planning for clinicians. But FSI computations can take several days to complete depending on the computational power available. In addition, carrying out these computations needs an expert in fluid dynamics and programming to interpret the simulation results for the clinician. Clinicians are often unable to obtain this kind of expertise or do not have the lead time to go through the detailed process to assess the prognosis of AAA in a particular patient. CFD is crucial in understanding the mechanics of aneurysm formation and rupture but it needs to be used in conjunction with other methods that can be faster than CFD to obtain a conclusion quickly. This can be done by the use of machine learning methods.

A summary of the machine learning method has been described before. It uses a combination of statistical methods, probability and optimization methods, to predict the direction of movement of a system. A “learning” model is created with the available retrospective data. A large quantity of such retrospective data is available at hospitals and this is helpful in developing learning models that can make prospective predictions having “learnt” from retrospective data. There are numerous statistics based machine learning methods that can be used along with CFD simulations so that more accurate and faster conclusions can be drawn for clinicians [78].

Data mining is a popular method used to derive conclusions in hemodynamic simulations. Kolachalama et al. [79] proposed a data mining technique that accounted for the geometric variability in patients for predicting cardiovascular flows. A Bayesian network-based algorithm was used to understand the influence of key parameters through a sensitivity analysis. Although Monte Carlo methods are suitable for output statistical analysis, the Bayesian approach brings out the relationships between the parameters using a multivariate approach. They tested the method on the human carotid artery bifurcation. A range of automated geometries were created for steady state 3D flow analysis. After CFD analysis was carried out, the output maximum wall shear stress was approximated as a function of the geometric variables. The data from the runs was used as a training data set to build the Bayesian model. A probability plot of the maximum wall shear stress could then be generated.

Geometric parameters (especially diameter of the aneurysm) are critical in defining the risk of rupture and have undergone most investigation in the field of data mining. The variation of geometric parameters amongst patients gives an opportunity for models to be developed to predict the characteristics of disease progression. Martufi et al. [80] carried out a geometrical characterization of the wall thickness distribution in AAA. They were able to train a model to differentiate the wall thicknesses in ruptured and unruptured AAA. The thickness difference was seen to be 7.8% between ruptured and unruptured AAA. This could be an important parameter to determine the risk rupture and plan early intervention.

This work has been extended by Shum et al. [81] who developed a model from 66 ruptured data sets and 10 nonruptured data sets and their geometric indices and wall thickness variations. The results of this study showed that, in addition to maximum diameter, sac length, sac height, volume, surface area, bulge height, and ILT volume were all highly correlated with rupture status. It was also observed that parameters such as volume of ILT were also highly correlated with rupture in addition to the size of the AAA. In this study, the overall classification accuracy was 86.6%. It used a decision tree algorithm that is one of the possible machine learning methods that can be used for large data sets requiring a decision output.

The above mentioned studies have been limited by the use of geometric parameters and, in particular, the maximum diameter of lumen alone as factors contributing to the rupture of an AAA. But other parameters such as patient history and comorbidities and presence of stents or other geometric parameters such as the aneurysm neck angles, tortuosity, or even genetic factors could well play a significant part in contributing to the determination of rupture risk of an AAA. Here, machine learning methods in parallel with CFD can be used to develop a tool that can be of practical use to clinicians as a predictive tool in the management of an AAA.

## 4. Conclusions

This review of the computational methods used in the prediction of rupture risk in AAA reveals that several aspects of the problem need to be understood before computational methods can be used to reliably predict rupture. These include the boundary conditions and material properties that weave in the patient specificity of the problem to be solved. These parameters are difficult to obtain in real clinical situations and have been approximated to a large extent in the literature. A suitable combination of these properties could lead to better prediction of the risk of rupture. But, given that measurement of boundary conditions and the material properties of the aortic wall in a particular patient is a very invasive process, computational methods have played a very big role in the biomechanical analysis of AAA. The combination of CFD and FEM has led to understanding of the flow pressure distribution, wall shear stress quantification, and effect of material properties and geometrical parameters. Computational methods have made patient-specific analyses possible, a feature essential for understanding the progression of AAA in a particular patient. Each patient has their own unique anatomy and pathophysiology that affects material properties and boundary conditions that can influence their management significantly.

Unfortunately, CFD/FEM methods, whilst providing crucial information on the pathophysiological mechanics of the aneurysm, can be very time consuming to undertake. This is made worse by the fact that FSI is a computationally expensive and complex method for routine use in real-life clinical situations. A much simpler interface therefore needs to be developed wherein the vascular clinician is in a position to assess the prognosis of the patient, rupture risk of the aneurysm and proceed to planning surgical, endovascular, or conservative treatments custom-made for that patient. This can be possible through the use of machine learning methods. The use of machine learning models in parallel with FSI computations can bridge the gap between translational and outcome research, thereby improving healthcare delivery and saving lives in aortic aneurysms.

## Figures and Tables

**Table 1:** Wall thickness measurements as reported in literature [52].

Author	Reported thickness (mm)	Method of measurement	Remarks
Di Martino et al., 2006 [53]	Elective AAA 2.5 ± 0.1 Ruptured AAA 3.6 ± 0.3 Mean 2.9	Optical (laser)	Thickness is inversely correlated with local strength; only anterior wall tested; use of laser measurement eliminates compression due to caliper

Raghavan et al., 2006 [54]	Min 0.23 Max 4.26 Median 1.48	Caliper	No discernible difference in thickness for small and large aneurysm; thickness slightly lower in posterior and right walls; thickness low in ruptured aneurysm near site of rupture

Thubrikar et al., 2001 [55]	Posterior 2.73 ± 0.46 Lateral 2.52 ± 0.67 Anterior 2.09 ± 0.51	Customized micrometer with resistivity meter	Thickness decreases from posterior to lateral to anterior walls; accuracy 0.05 mm

**Table 2:** Comparison of parameters investigated using FSI methods.

Reference	Method	Parameter	Remarks
Di Martino et al., 2001 [65]	FSI (ALE)	Maximum pressure	First accurate calculation of FSI

Scotti et al., 2008 [72]	FSI	Wall shear stress	20% more WSS if FSI method is used

Scotti et al., 2005 [73]	FSI	Asymmetry and wall thickness	Varying wall thickness and asymmetry increases von Mises stress

Li and Kleinstreuer, 2007 [74]	FSI	Neck angle, asymmetry, and bifurcation angle	Large neck angle leads to elevated von Mises stress; lateral asymmetry has higher stress
